# Surgical resection of malignant tumors at the ischial tuberosity: a new surgical incision design and clinical experience

**DOI:** 10.3389/fonc.2025.1578468

**Published:** 2025-05-09

**Authors:** Haocheng Cui, Kai Zheng, Ming Xu, Qian Chen, Kai Zhai, Xiuchun Yu

**Affiliations:** Orthopedic Department, 960 Hospital of People’s Liberation Army, Jinan, Shandong, China

**Keywords:** ischial tuberosity, malignant tumor, osteotomy, surgical incision, clinical application

## Abstract

**Objective:**

This study aims to present the anatomical characteristics of a novel surgical incision designed for single ischiectomy and to share our practical experience with its application.

**Methods and materials:**

The newly developed surgical incision begins 5–6 cm below the posterior superior iliac spine and extends toward the ipsilateral greater trochanter, passing through the ischial tuberosity. It is then continued distally along the femur via the greater trochanter, forming what is referred to as a PIG-S incision. This study outlines the surgical resection procedures in two patients diagnosed with ischiotuberous chondrosarcoma who underwent surgery using the PIG-S incision. Key outcomes include surgical duration, intraoperative blood loss, and postoperative complications.

**Results:**

In one case, the patient experienced tumor recurrence nine months after an initial curettage of the ischial tubercle, necessitating complete tumor resection via the PIG-S approach. The second patient presented with ischiotuberous chondrosarcoma involving the lower margin of the acetabulum. A combined surgical approach using a PIG-S incision and an anterior ilioinguinal incision was adopted for tumor resection in regions II and III, with pelvic reconstruction performed using a 3D-printed prosthesis. The surgeries lasted for the two patients 90 and 150 min, with intraoperative blood losses of 1000 and 2000 mL, respectively. No postoperative incision-related complications were observed in either patient.

**Conclusions:**

The PIG-S incision allows comprehensive exposure of crucial structures within the sciatic region, facilitates complete resection of the ischial tuberosity, and maximally preserves the sciatic nerve.

## 1 Introduction

The ischial tuberosities are bilateral bony prominences adjacent to the anus, aligned horizontally with the lesser trochanters of the femur. Deeply positioned near the sciatic nerve, these structures serve as attachment points for the hamstring muscles, including the long head of the biceps femoris, semitendinosus, and semimembranosus, as well as the sacrotuberous ligament. The ischial tuberosity is a common site for various primary bone tumors, such as chondrosarcoma, giant cell tumors, and aneurysmal bone cysts (1–3). Traditionally, a posterior ischial incision has been utilized for tumor curettage or subperiosteal excision. This incision starts below the posterior inferior iliac spine and follows the hip crease laterally, passing through the ischial tuberosity. It can also be employed for open reduction and internal fixation in ischial tuberosity fractures (4). However, in the case of malignant tumors, extensive ischial bone resection is often required, rendering the posterior ischial incision inadequate. A combined ilioinguinal or transperineal approach is often necessary, depending on the tumor’s size and location.

According to Enneking’s classification of pelvic tumor regions, the acetabulum is in Region II, while the ischium and pubis fall under Region III (5). Region II resection usually involves a combination of anterior and posterior approaches, such as an ilioinguinal incision paired with a lateral incision extending posterior to the greater trochanter and perpendicular to the iliac spine (6). Region III resection typically encompasses the pubic symphysis, subpubic rami, and lateral rami extending to the superior pubic rami and medial acetabulum. In such cases, a vertical incision from the pubic tubercle along the ilioinguinal line is often used to expose the subpubic rami and ischial tuberosity (7).

Guo et al. (8) reported successful resection of a giant cell tumor of the pelvic bone involving Regions II and III using a combined ilioinguinal and posterior ischial incision, followed by pelvic ring reconstruction using a 3D-printed prosthesis. They concluded that this combination is effective for managing tumors spanning Regions II and III. When tumors are located deep within the inferior pubic ramus or ischial tuberosity, a paraperineal approach may also be required (9).

While most documented surgical incisions address tumors in Region III or those spanning Regions II and III, there remains a lack of guidance on optimal incision strategies for malignant tumors confined to the ischial tuberosity. Complete tumor resection in this area necessitates precise osteotomies at the lesser sciatic notch and ischial ramus while preserving the integrity of the sciatic nerve. The ilioinguinal incision is advantageous for exposing the superior and inferior pubic rami but offers poor access to the lesser sciatic notch. Conversely, the paraperineal incision allows good access to the ischial ramus and tuberosity but limits osteotomy at the lesser sciatic notch. The posterior ischial incision permits osteotomy at the notch but does not adequately expose the ischial ramus, increasing the risk of sciatic nerve injury (10).

To address these limitations, we designed a novel surgical approach tailored specifically for the total resection of malignant tumors localized to the ischial tuberosity. Based on the clinical data from two cases of ischiotuberous chondrosarcoma treated using this new approach, this study systematically presents the incision design, relevant anatomy, and operative procedures. Objectives: (1) To evaluate the necessity and clinical implications of complete resection for malignant tumors confined to the ischial tuberosity; (2) To propose and introduce a novel surgical incision design; (3) To analyze the anatomical considerations and procedural techniques associated with this new approach.

## 2 Materials and methods

### 2.1 Anatomical studies

**Figure 1:**
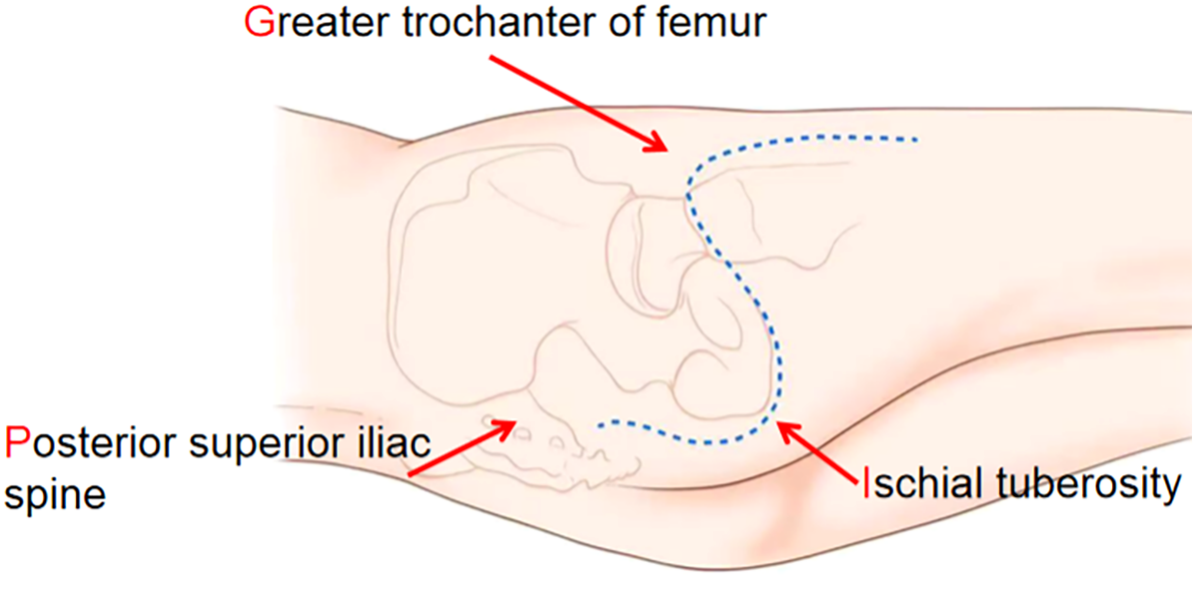
The incision for the ischial tubercle resection started 5–6-cm below the posterior superior iliac spine, curved around the greater trochanter via the ischial tubercle, and extended distally along the femoral greater trochanter. This incision is called the PIG-S incision.

Design of the incision for total excision of the ischial tuberosity: The incision was created at approximately 5–6-cm inferior to the posterior superior iliac spine and followed a curvilinear path around the Ischial tuberosity, directed toward the Greater trochanter of the ipsilateral femur. It subsequently extended distally along the limb (**Figure 1**). The incision was designed based on the surface projections of several key anatomical landmarks. The initial letters and the approximate shape of this incision were adopted (hereinafter referred to as the PIG-S incision).

**Figure 2:**
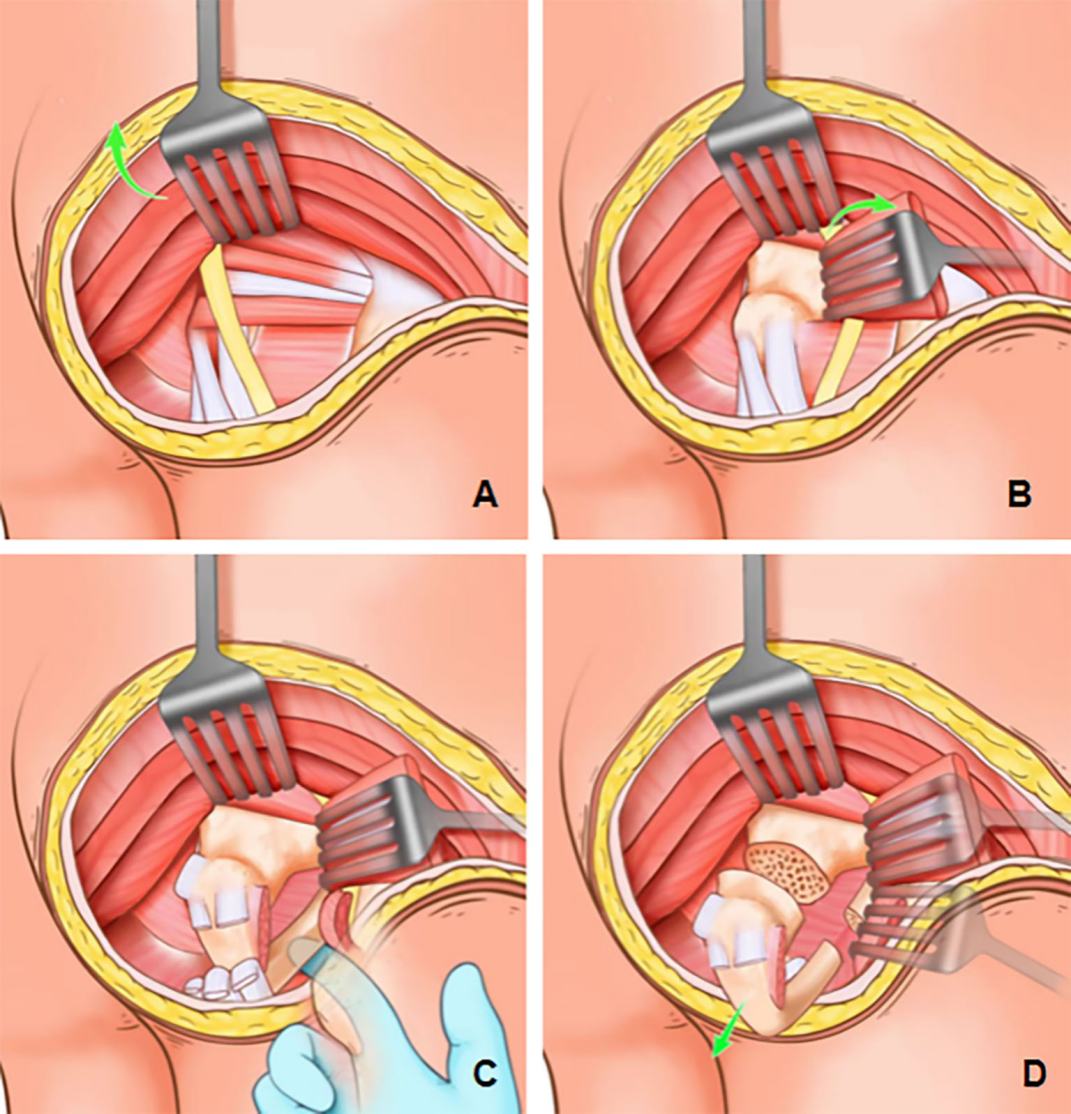
PIG-S incision exposure process. **(A)** The lower margin of the gluteus maximus was exposed, and a partial section of the gluteus maximus insertion was performed. The muscle could be retracted or elevated to expose the underlying anatomical structures. **(B)** Under the premise of protecting the sciatic nerve, the external rotator muscle group was incised and the quadratus femoris muscle was sectioned. **(C)** Finger was carefully inserted into the space between the ischial tuberosity and the lesser trochanter of the femur to palpate and identify the course of the ischial ramus. **(D)** The ischial tuberosity was completely removed by osteotomy at the ischial ramus and lesser sciatic notch.

The primary anatomical structure exposed through the PIG-S incision is the gluteus maximus muscle. This approach provides clear visualization of the majority of the muscle’s inferior border and part of its insertion. After partially sectioning the insertion of the gluteus maximus, the muscle can be retracted or elevated to expose the underlying anatomical layers. Deeper muscles exposed include the superior gemellus, obturator internus, inferior gemellus, quadratus femoris, and the hamstring muscles. The sciatic nerve, the predominant neural structure in the gluteal region, traverses from the superficial plane of the short external rotators to the deep surface of the biceps femoris. With careful protection of the sciatic nerve, the external rotator muscles are incised, followed by the sectioning of the quadratus femoris. At this point, a finger can be carefully inserted into the space between the ischial tuberosity and the lesser trochanter of the femur to palpate and trace the course of the ischial ramus. Subsequently, the hamstring tendon attachments to the ischial tuberosity and the sacrotuberous ligament are carefully transected. Dissection continues using a finger dissector to access the deep surface of the ischial tuberosity, enabling precise osteotomy of the ischial ramus. Complete excision of the ischial tuberosity is then accomplished through meticulous osteotomy at the level of the lesser sciatic notch (**Figure 2**).

### 2.2 Case presentation

#### 2.2.1 Case 1

**Figure 3:**
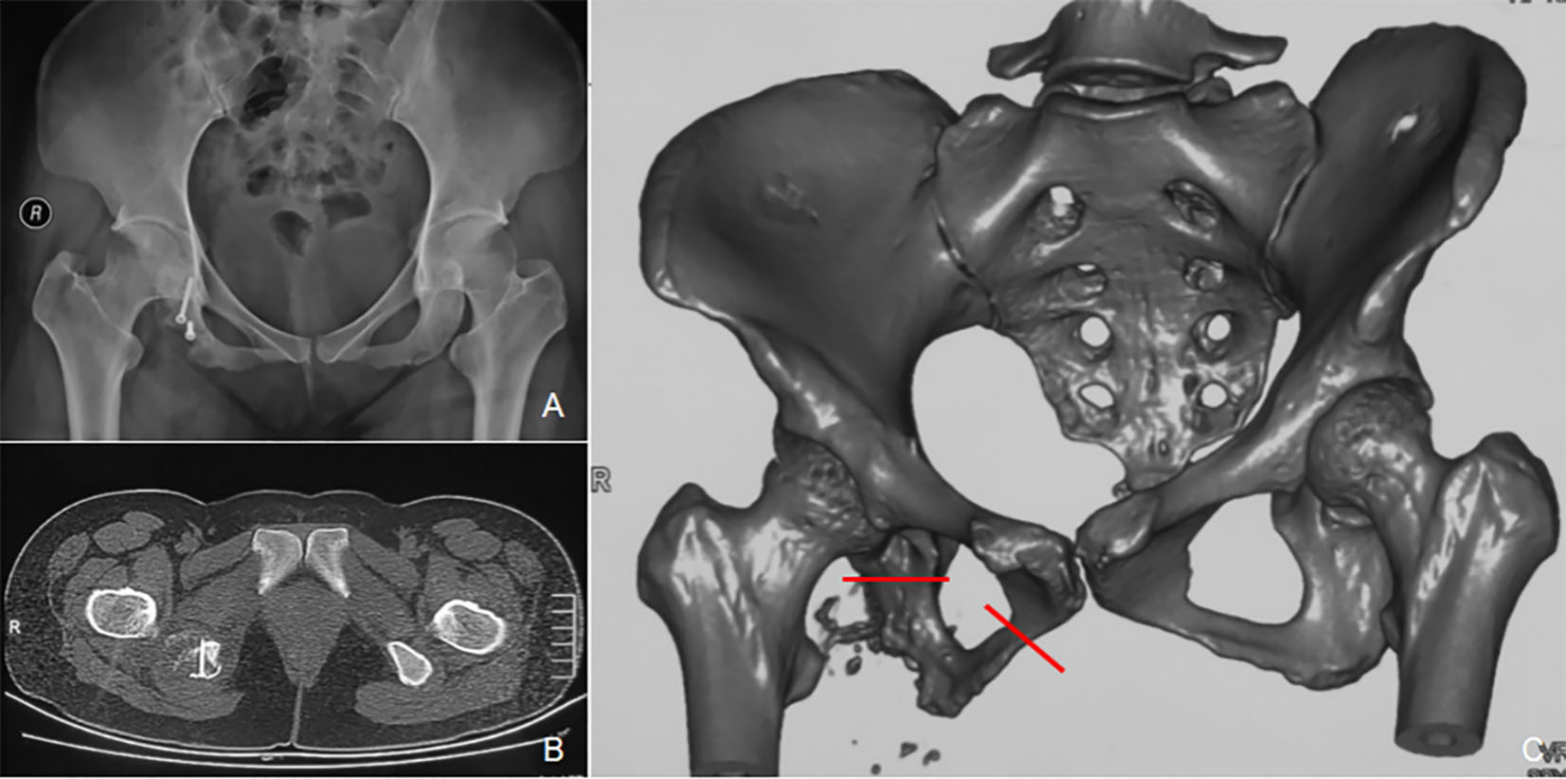
A 31-year-old female patient with a recurrence of right ischiatic tuberous chondrosarcoma after curettage. **(A)** Pelvic radiographs displaying that internal fixation was in place and that the right ischiatic tubercle was lacking in the bone and soft tissue mass around it. **(B)** CT scans revealed lytic bone destruction of the right ischiatic tubercle with soft tissue mass and punctate calcification. **(C)** Preoperative plan: An extended resection of the right ischiatic tubercle was proposed (the red line indicates the intraoperative osteotomy line drawn up before surgery).

A 31-year-old woman was diagnosed with a right ischial bone tumor and initially underwent curettage, bone grafting, and internal fixation at a local hospital. Postoperative histopathology confirmed grade II chondrosarcoma. Nine months later, the patient reported recurrent pain in the right buttock. At physical examination, a healed posterior ischial incision was noted on the right buttock, with normal local skin color and temperature, but significant tenderness over the ischial tuberosity. Pelvic radiographs revealed a bone defect in the right ischial tuberosity with notable osteolytic destruction. CT scans confirmed bone resorption and the presence of a soft tissue mass with ill-defined margins. Biopsy confirmed recurrent chondrosarcoma. An extended resection of the right ischial tumor was performed at our institution (**Figure 3**).

##### 2.2.1.1 Operation

**Figure 4:**
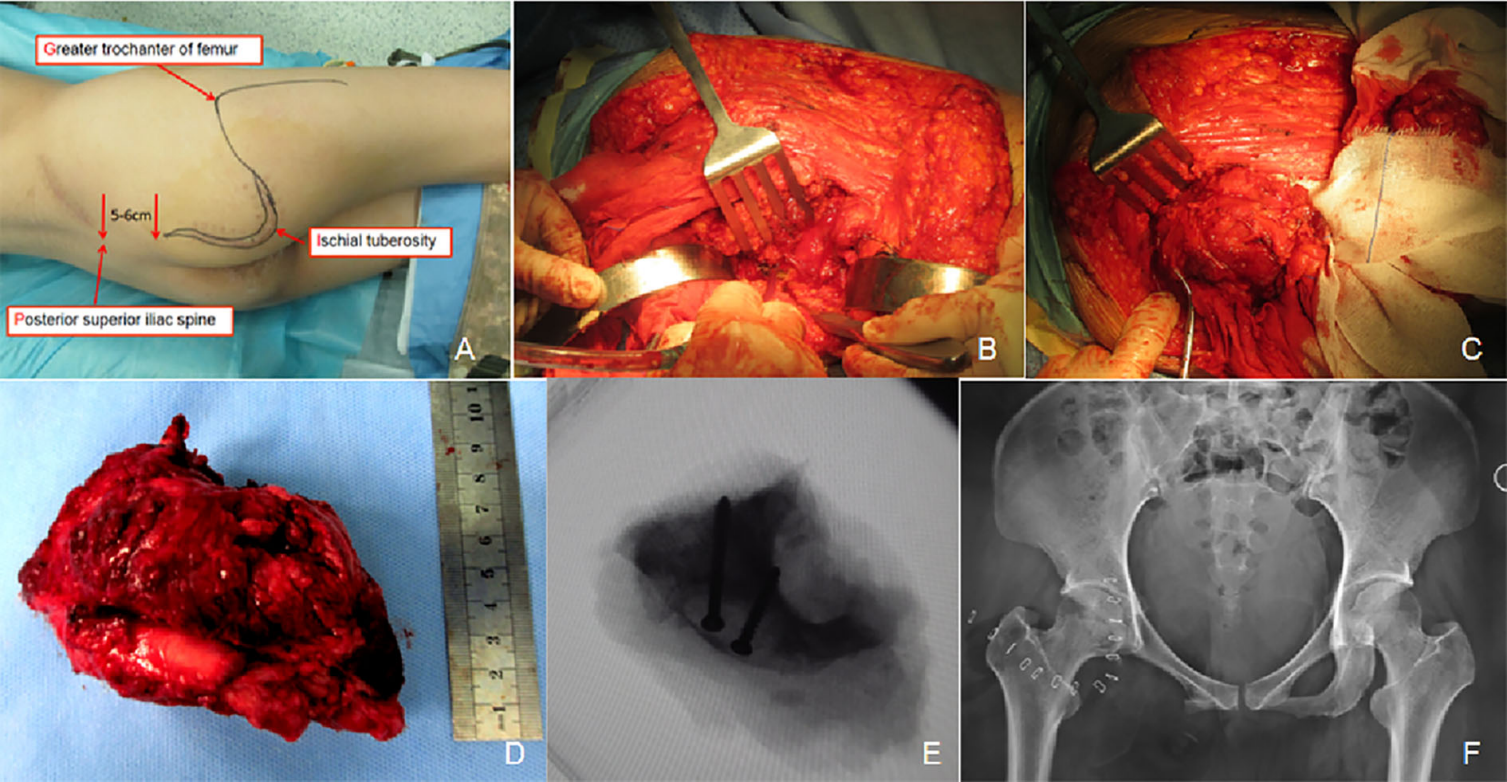
The PIG-S incision was performed to accomplish the resection of the ischial tubercles. **(A)** Patient positioning and incision marking. **(B)** Osteotomy of the ischial and subpubic rami was performed. **(C)** Osteotomy at the lesser sciatic notch was conducted. **(D)** The entire specimen of tumor tissues from the ischial tubercle was excised *en bloc*. **(E)** Intraoperative fluoroscopy confirmed that the two osteotomy lines were consistent with the preoperative planning. **(F)** Postoperative radiographs demonstrated satisfactory resection of the tumor tissues.

Under general anesthesia, the patient was placed in the left lateral decubitus position, with the torso inclined forward at an approximate angle of 45°. A PIG-S incision encompassing the previous surgical scar was made. Layer-by-layer dissection exposed the posterior margin of the gluteus maximus, which was sectioned at its femoral insertion and retracted proximally. The sciatic nerve was identified and protected. The interspace between the external rotator muscles and the obturator internus was incised, and fingers were used to palpate the ischial branches between the lesser trochanter and the ischial tuberosity. The quadratus femoris, long head of the biceps femoris, semitendinosus, semimembranus, and sacrotuberous ligament were transected. Attachments of the adductor maximus on the ischial rami were also severed. After elevating the periosteum with a periosteal elevator, osteotomy was performed at the junction of the ischium and subpubic ramus. A second osteotomy was made at the lesser sciatic notch, enabling complete resection of the ischial tuberosity tumor. Intraoperative blood loss was approximately 1000 mL. The patient received 400 mL of packed red blood cells and 400 mL of plasma. Postoperative pathology confirmed grade II chondrosarcoma with surrounding soft tissue involvement, but no neoplastic cells were identified at the resection margins (**Figure 4**).

##### 2.2.1.2 Follow-up

At an 8-month follow-up after the second operation, pelvic CT indicated an irregular high-density shadow in the soft tissue mass at the right ischial tuberosity surgical site. MRI suggested a mass with low T1 and high T2 signals at the same site, which invaded the posterior margin of the acetabulum and the right hip joint. Biopsy confirmed recurrence of chondrosarcoma at the right ischial tuberosity. The patient underwent extended tumor resection and femoral head replacement. At the 2-month follow-up after the third operation, lung CT identified multiple bilateral pulmonary nodules. By 5 months, the tumor had recurred locally, invading the right pelvis and proximal femur. Lung CT confirmed pulmonary metastases consistent with chondrosarcoma. The patient was treated with oral apatinib mesylate and was subsequently lost to follow-up.

#### 2.2.2 Case 2

**Figure 5:**
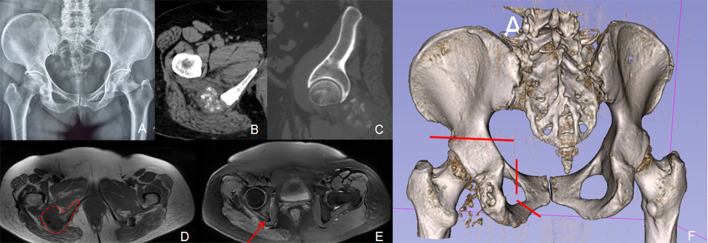
A 54-year-old woman complained of pain in her right buttock. A preoperative puncture biopsy confirmed the diagnosis of chondrosarcoma involving the right ischial tuberosity and the posterior margin of the acetabulum. **(A)** Pelvic radiographs displayed abnormal bone in the right ischial tuberosity and the lower margin of the acetabulum, with patchy bone sclerosis and low-density areas visible within. **(B, C)** CT scans indicated popcorn-like bone abnormalities in the right ischial tuberosity, involving a portion of the lower margin of the acetabulum. **(D, E)** MRI scans showing a mass-like lesion with mixed low T1 and high-low T2 signals in the right ischial tuberosity (encircled by the red dotted line), while the posterior margin of the right acetabulum was involved (indicated by a red arrow), with patchy edema reaction bands visible in the surrounding muscles and soft tissues. **(F)** Preoperative plan: Intended to perform *en-bloc* resection of the ischial and acetabular regions (the red line represents the proposed intraoperative osteotomy line).

A 54-year-old woman presented with over a month of right buttock pain. At physical examination, the patient exhibited mild swelling in the right buttock, with marked tenderness at the ischial tuberosity. The local skin color and temperature were normal. Right hip joint mobility was mildly restricted. Pelvic radiographs showed abnormalities in the right ischial tuberosity and acetabular inferior margin, with lamellar osteosclerosis and areas of low bone density and continuous cortical bone. CT revealed “popcorn-like” calcification, suggestive of chondrosarcoma, involving part of the acetabular margin. MRI showed heterogeneous signals in the ischial region and edema in the surrounding muscle and soft tissues. Puncture biopsy confirmed grade 3 chondrosarcoma. Surgical resection of the tumor involving the right ischium and acetabulum was planned, followed by reconstruction with a 3D-printed pelvic prosthesis (**Figure 5**).

**Figure 6:**
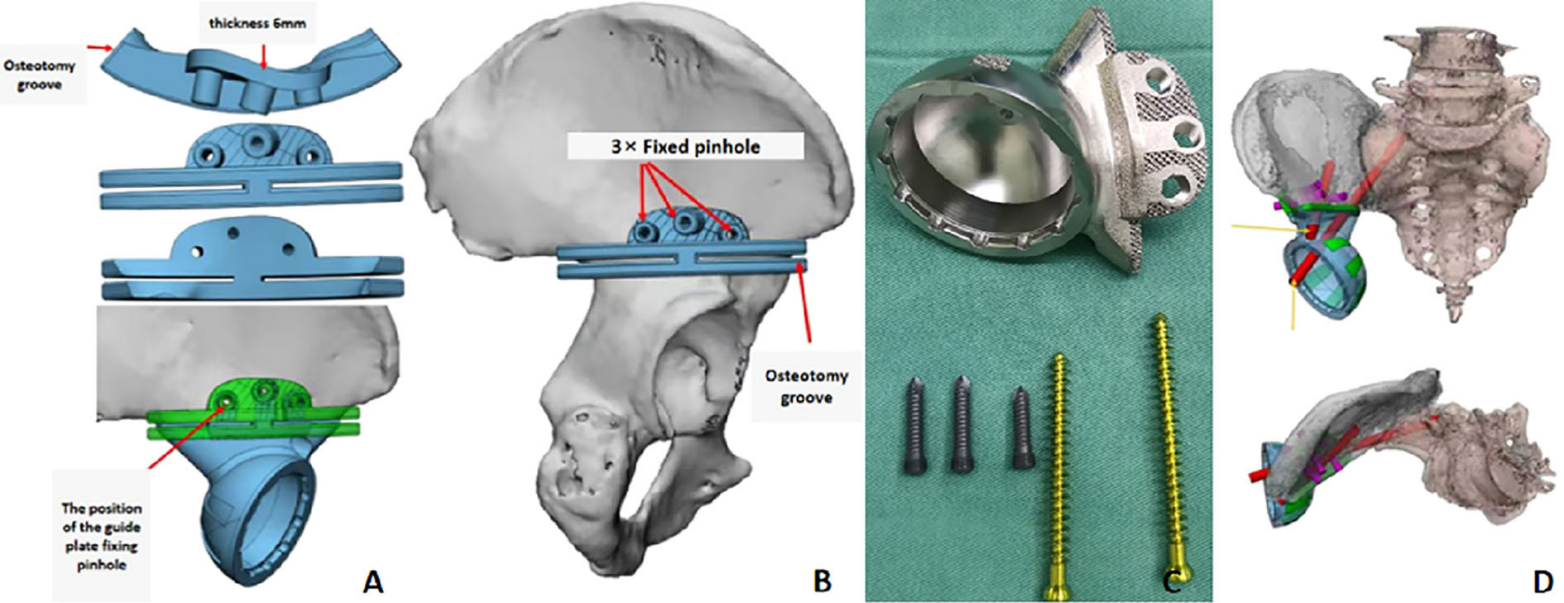
Preoperative osteotomy and prosthesis design plan. **(A)** The 3D printed acetabular upper osteotomy guide plate is made of nylon. The position of the guide plate fixation pin hole corresponds to that of the screw on the outer wing of the prosthesis. **(B)** The placement position of the osteotomy guide plate is located at the upper edge of the acetabulum, adhering to the bone surface along the upper edge of the acetabulum. **(C)** The customized acetabular prosthesis has a 3D-printed grid-like void on the bone contact surface to facilitate bone ingrowth. Three short nails are fixed to the outer plate of the ilium, and two long nails are driven into the acetabular cup, with one of them passing through the sacroiliac joint. **(D)** After the installation of the customized acetabular prosthesis is completed, the rotational center of the hip joint can be anatomically reconstructed *in situ*.

Before surgery, pelvic data of the patient were obtained through CT scanning to reconstruct a 3D model of the patient’s pelvis. Using this model, a 3D-printed osteotomy guide plate was designed to facilitate precise osteotomy along the superior edge of the acetabulum. Based on the defined osteotomy boundaries, a patient-specific acetabular prosthesis was fabricated using 3D printing technology. This digitally planned reconstruction enabled accurate anatomical reconstruction of the hip joint’s rotational center (**Figure 6**).

##### 2.2.2.1 Operation

**Figure 7:**
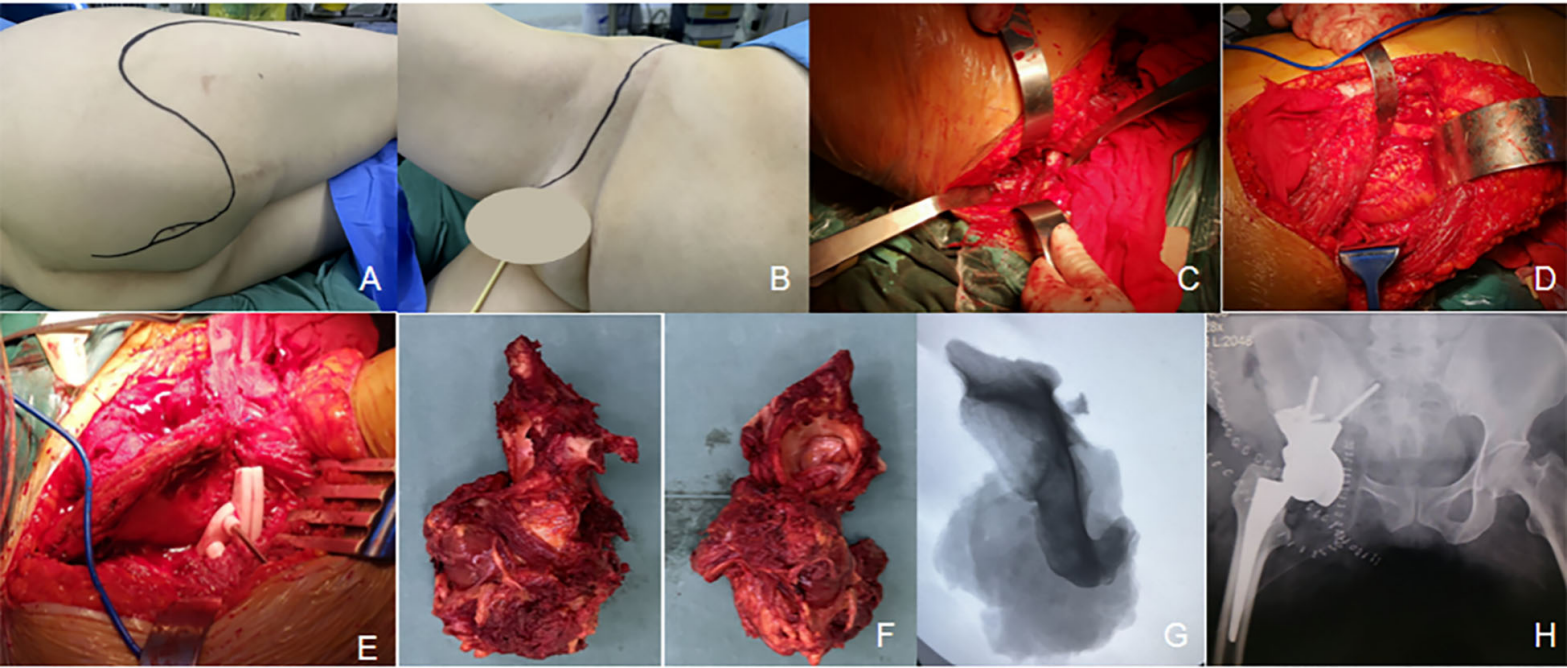
The resection of ischiatic tuberosity and acetabular tumors was performed by using a combined PIG-S incision and ilioinguinal incision approach. **(A)** Patient in the swing position with posterior PIG-S incision. **(B)** Anterior ilioinguinal incision. **(C)** The anterior incision exposed the superior pubic ramus, allowing precise osteotomy. **(D)** The posterior incision facilitated access to the gluteus maximus while protecting the sciatic nerve. **(E)** An osteotomy guide was installed above the acetabulum to guide the osteotomy procedure. **(F)** *En-bloc* resection of the acetabular and ischiatic tumor specimens was achieved. **(G)** Intraoperative fluoroscopy confirmed satisfactory resection, with the osteotomy line aligning with the preoperative plan. **(H)** Postoperative X-rays demonstrated optimal positioning of the 3D-printed prosthesis.

The patient was positioned in the left lateral decubitus position with a slight forward tilt. A combined anterior and posterior approach was used: the anterior approach via an ilioinguinal incision and the posterior approach via a PIG-S incision. The anterior incision exposed the muscle attachments around the anterior superior iliac spine. The rectus femoris and femoral vessels were carefully retracted to fully expose the superior pubic ramus, which was then transected as per the preoperative plan. Next, the posterior PIG-S incision was made. The proximal femoral insertion of the gluteus maximus was released to allow exposure and protection of the sciatic nerve. A tumor mass was identified inferior to the sciatic nerve. To access the mass, the semitendinosus, semimembranosus, long head of the biceps femoris, and the sacrotuberous ligament were transected. The muscular portion of the adductor maximus and the external rotators, including the obturator internus, were also carefully dissected. A finger was inserted between the lesser trochanter and the ischial tuberosity to accurately trace the ischial rami. The deep soft tissue mass was sharply dissected, and osteotomy was performed at the junction of the ischial and pubic rami. After incising the joint capsule, the hip joint was dislocated, and the femoral neck was transected 1.5 cm proximal to the lesser trochanter. The femoral head was subsequently excised. Using the osteotomy guide plate, precise osteotomy of the posterior superior acetabular margin was performed. The tumor was completely resected in an anterior-to-posterior direction. A customized 3D-printed acetabular prosthesis and a femoral stem prosthesis with a ceramic ball head were implanted to reconstruct the hip joint. The hip capsule was reconstructed using a standard surgical mesh, and surrounding muscles were sutured to the mesh. Intraoperative C-arm fluoroscopy confirmed satisfactory prosthesis positioning. The resected tumor specimen matched the preoperative plan (**Figure 7**). Postoperative pathological: High-grade chondrosarcoma (Grade III). No tumor was found at the bone end or the soft tissue margin. Estimated intraoperative blood loss was approximately 2000 mL. The patient received 800 mL of pre-stored autologous blood, 1200 mL of packed red blood cells, and 350 mL of plasma.

##### 2.2.2.2 Follow-up

The patient underwent regular follow-up and was monitored up to the 15th month postoperatively. At 6 months, radiographs revealed breakage of the screw across the sacroiliac joint, though prosthesis positioning remained optimal. CT demonstrated good integration between the 3D-printed acetabular prosthesis and the ileum. The patient was ambulating with crutches and had a satisfactory function, with a Musculoskeletal Tumor Society (MSTS) (11) score of 20. At the 15-month follow-up, radiographs again showed a broken sacroiliac screw but no change in prosthesis positioning. Pelvic CT confirmed stable integration of the prosthesis, and lung CT showed no abnormalities. The patient was ambulating independently and had regained good lower limb function, with an MSTS score of 24.

## 3 Discussion

### 3.1 The imperative of *en bloc* resection in pelvic chondrosarcoma

Chondrosarcoma constitutes approximately 20% of primary malignant bone tumors and ranks third after myeloma and osteosarcoma. It predominantly affects middle-aged individuals and is characterized by the production of a cartilage matrix by tumor cells (12). The pelvis is the most frequently involved site, with the ilium being the most commonly affected region, followed by the pubis and ischium (1). Although chondrosarcoma generally exhibits slow growth and low metastatic potential, it is largely resistant to radiotherapy and chemotherapy. Therefore, surgical resection remains the cornerstone of treatment. While the overall prognosis is favorable, incomplete resection is associated with a high risk of local recurrence (13).

Ozaki et al. (14) compared outcomes in 26 patients with chondrosarcoma who underwent intralesional curettage with those in 38 patients with chondrosarcoma who received wide or marginal resection. The 20-year recurrence-free survival rate was only 7% in the curettage group compared with 64% in the wider resection group. Similarly, Streitburger et al. (15) analyzed clinical data from 80 patients with low-grade chondrosarcoma, concluding that while intralesional curettage might be acceptable for extremity lesions, marginal or wide resection is necessary for pelvic involvement to prevent tumor recurrence. Although local recurrence may not significantly reduce overall survival, it increases the likelihood of tumor progression to higher-grade chondrosarcomas, thereby elevating the risk of metastasis and ultimately reducing long-term survival (2).

In Case 1, tumor recurrence occurred 9 months after curettage. The second operation involved en bloc resection of the ischial region. Eight months after the operation, the tumor recurred. The third surgery included extensive circumferential resection of the acetabulum and femoral head exclusion. Five months after the operation, the tumor recurred and progressed with lung metastasis. By contrast, Case 2 underwent wide resection of the ischial and acetabular regions based on a definitive biopsy diagnosis and remained recurrence-free after 15 months. These cases highlight that complete, *en bloc* resection of chondrosarcoma, especially when located at the ischial tuberosity, is essential to achieving favorable long-term outcomes.

### 3.2 The design philosophy and application scope of PIG-S incision

Pelvic tumors are generally challenging to detect at an early stage due to their deep anatomical location. Consequently, most patients present with tumors significantly larger than those typically found in the limbs (16, 17). Consequently, achieving complete resection often necessitates a relatively large or combined surgical approach. Enneking and Dunham advocate for the ilioinguinal (extended inguinal) approach as the standard for treating pelvic tumors (5). Originally designed by Letuoumel (18), this approach is primarily used for the reduction and fixation of anterior wall fractures of the acetabulum, anterior column fractures, and certain transverse fractures. The incision begins at the pubic tubercle, extends along the inguinal ligament to the anterior inferior iliac spine, and continues along the iliac crest to the posterior superior iliac spine. For anterior (Region III) resections, the incision must be extended medially toward the inner thigh, while posterior (Region I) resections may require posterior extension to the spinal midline. If necessary, a lateral incision can be added to access the posterior acetabulum, ischium, and sciatic nerve.

Although this mature surgical approach allows for the resection of most tumors in Regions II and III and facilitates prosthetic reconstruction, it presents significant challenges in achieving marginal resection of tumors located posterior to the acetabulum and ischium. The approach is associated with a high degree of operational blind spots and an increased risk of injury to nearby blood vessels, nerves, and organs. Especially, when the aim is to preserve the hip joint or a part of it and perform limited reconstruction, the hip joint itself obstructs access to posterior tumors, rendering safe marginal resection of the tumor extremely difficult and risky (19). Therefore, *en bloc* resection of ischial tuberosity tumors cannot be effectively achieved through the ilioinguinal approach alone.

Although the posterior ischial incision was developed for ischial tuberosity tumors, it provides limited exposure, carries a risk of sciatic nerve injury, and does not adequately expose the ischial ramus (20).

Upon reviewing the clinical courses of two patients discussed earlier, we observed a notable distinction from other large pelvic tumors: tumors located near the ischial tuberosity—a critical weight-bearing region during sitting—may elicit pain symptoms earlier in their progression. This early discomfort often prompts patients to seek medical attention sooner, enabling earlier diagnosis. If *en bloc* resection is performed while the tumor remains small, the prognosis can be significantly improved. Additionally, in cases where the tumor is confined to Region III, reconstruction may not be necessary following resection (21).

In response to these findings, we designed the PIG-S incision to facilitate *en bloc* resection of malignant tumors in the ischial tuberosity region. By extending the traditional posterior ischial incision medially and laterally, we can fully expose the inferior border of the gluteus maximus and its femoral insertion, while also safely identifying and preserving the sciatic nerve. Although this incision is relatively extensive, it does not significantly increase intraoperative bleeding or surgical duration compared with the ilioinguinal or combined approach. In Case 2, where acetabular prosthesis reconstruction was performed, intraoperative blood loss was 1000 and 2000 mL in the two surgeries, with durations of 90 and 150 min, respectively. Postoperative follow-up revealed no complications such as poor wound healing or infection. Therefore, we compare the PIG-S incision with other classic posterior lateral pelvic incisions (**Table 1**) and preliminarily propose that the PIG-S incision is applicable in the following situations: 1. Isolated malignant tumors of the ischial tuberosity, especially chondrosarcoma; 2. Tumors not involving the sacral plexus, or cases where preservation of the sciatic nerve function is critical; 3. Complex tumors involving zones II/III that require combined approaches for complete resection. However, for patients with extensive soft tissue involvement, multiple metastases, or severe comorbidities who cannot tolerate lengthy operations, the PIG-S incision should be employed with caution.

**Table 1:** Comparison of surgical incision characteristics in pelvic regions II and III.

Characteristics of the approach	PIG-S approach	Ilioinguinal approach	Iliofemoral approach(S-P)^a^	Posterior acetabular approach(K-L)^b^	Lateral-perineal approach	Posterior ischial incision
Exposed regions	II(posterior acetabular)+III(ramus of ischium)	I+II(Anterior acetabulum)+III(Ramus superior ossis pubis )	I+II(Anterior acetabulum)	I+II(posterior acetabular)	III(ramus of ischium)	III(ischial tuberosity)
Important anatomical structures	ischial tuberosity+lesser sciatic notch.+ischial ramus+ischiadic nerve	ilium+Femoral arteries and veins+femoral nerve	Femoral arteries and veins+femoral nerve+greater sciatic notch	posterior acetabular	remi inferior ossis pubis +ramus of ischium	ischial tuberosity
exposure of nerve	Direct visualization and protection of the sciatic nerve throughout its course	Prone to damage femoral lateral cutaneous nerve and femoral nerve	Protect the femoral nerve, Prone to damage femoral lateral cutaneous nerve	prone to damage the sciatic nerve	Prone to injury of obturator nerve	prone to damage the sciatic nerve
Vital blood vessel	protect the blood vessels under the buttocks.	Avoid damage the Coronal Mortis	_	Prone to damage superior gluteal blood vessels	protect the perineal vessels	_
Dis-advantaged	Large incision	Large incision	Large incision	Large incision	susceptibility to infection	Insufficient exposure

a Smith-Petersen approach.

b Kocher–Langenbeck approach.

### 3.3 Anatomical and operational key points of PIG-S incision

The PIG-S incision can be used alone for complete resection of the ischial tuberosity or combined with other incisions for more extensive tumor removal. Key anatomical and operational features include the following:

#### 3.3.1 The gluteus maximus and the subgluteal space beneath it can be fully exposed

The gluteus maximus, the largest and most superficial muscle of the gluteal region, is broad with a thick contour and slightly quadrilateral in shape. It originates from the posterior superior iliac spine, iliac crest, lumbodorsal fascia, sacrotuberous ligament, sacral and coccygeal bones, and sacral ligaments. Its fibers run obliquely downward and outward, with the lower quarter inserting into the greater trochanter of the femur. The gluteus maximus bursa separates the tendon from the greater trochanter of the femur. The upper three quarters form a band-like aponeurosis that merges with the tensor fasciae latae aponeurosis to form the iliotibial tract, which descends and finally attaches to the lateral condyle of the tibia. The PIG-S incision basically follows the lower margin of the gluteus maximus and turns toward the greater trochanter at the ischial tuberosity. Once the femoral insertion of the gluteus maximus is detached, the muscle can be retracted proximally, allowing full exposure of the subgluteus space (22). This semi-enclosed anatomical region connects with the pelvic cavity through the greater sciatic foramen. Anteriorly, it is bordered by the iliac periosteum, piriformis, external rotator group, and joint capsule; superiorly, by the gluteal origins and iliac crest; inferiorly, by the gluteal insertions; and posteriorly, by the gluteus maximus itself. The fascia within the space is dense, and the sacrospinous and sacroiliac ligaments are structurally robust, acting as natural barriers to anterior tumor extension (23).

#### 3.3.2 The sciatic nerve can be fully exposed and protected

The sciatic nerve, the most critical structure in the subgluteus maximus space, emerges from the sacral plexus and exits the pelvis through the greater sciatic foramen. It is enveloped by a protective layer of adipose tissue. When dissecting the nerve proximally, the inferior gluteal artery and vein—located near the foramen—become visible and must be handled with care. Upon lifting the gluteus maximus, deeper structures including the piriformis, superior and inferior gemellus, obturator internus, and quadratus femoris are revealed. Resection of the superior and inferior gemellus, obturator internus, and quadratus femoris allows for posterior retraction of these muscles, effectively shielding and preserving the sciatic nerve.

#### 3.3.3 A single incision can be made for the lesser sciatic notch and ischial ramus osteotomy

Following the dissection of the external rotator muscles and the quadratus femoris, the lesser sciatic notch can be easily exposed proximally, allowing for osteotomy at this site. By inserting a finger into the space between the lesser trochanter of the femur and the ischial tuberosity, the deep surface of the ischial tuberosity can be palpated. After transecting the sacrotuberous ligament, along with the long head of the biceps femoris, semitendinosus, semimembranosus, and part of the adductor magnus at their insertion points, the junction of the inferior pubic ramus and ischial ramus becomes accessible. At this point, a wire saw or bone chisel can be used to perform the osteotomy. Once completed, the ischial tuberosity can be entirely removed. Continued proximal dissection of the flap allows exposure of the superior margin of the acetabulum. In Case 2, we accessed the upper edge of the acetabulum through the posterior PIG-S incision and performed a Region II osteotomy using a pre-positioned osteotomy guide plate.

## 4 Limitation

This retrospective study includes only two cases, as the implementation of a novel surgical approach requires informed patient consent and the accumulation of reliable, high-quality clinical data through long-term follow-up. As an initial exploration, this study is limited by the small sample size and absence of a control group. Future research should adopt a prospective design, include a control group, and expand the sample size. Additionally, since all case data originated from a single institution, future studies should aim for multi-center collaboration to enhance the generalizability of the findings.

## 5 Conclusions

The careful selection of a surgical incision is crucial for the successful resection of pelvic tumors. Adequate surgical exposure is essential; attempting tumor removal through a limited incision may increase the risk of damage to vital structures and compromise the quality of resection. Optimal surgical outcomes are strongly linked to appropriate surgical techniques and approaches. The PIG-S incision allows comprehensive exposure of key anatomical structures in the ischial region, enabling *en bloc* resection of the ischial tuberosity while effectively preserving the sciatic nerve function. Moving forward, we aim to further validate this approach through additional clinical cases, thereby contributing to the anatomical and clinical understanding of this surgical technique.

## Data Availability

The original contributions presented in the study are included in the article/supplementary material. Further inquiries can be directed to the corresponding author.
